# Injection Molding Micro‐ and Nanostructures in Thermoplastic Elastomers

**DOI:** 10.1002/mame.201600011

**Published:** 2016-05-06

**Authors:** John M. Stormonth‐Darling, Anwer Saeed, Paul M. Reynolds, Nikolaj Gadegaard

**Affiliations:** ^1^ Division of Biomedical Engineering School of Engineering University of Glasgow Rankine Building, Oakfield Avenue Glasgow G12 8LT UK

**Keywords:** elastomer, micro, molding, molding, nano

## Abstract

Flexible polymers such as poly dimethyl siloxane (PDMS) can be patterned at the micro‐ and nanoscale by casting, for a variety of applications. This replication‐based fabrication process is relatively cheap and fast, yet injection molding offers an even faster and cheaper alternative to PDMS casting, provided thermoplastic polymers with similar mechanical properties can be used. In this paper, a thermoplastic polyurethane is evaluated for its patterning ability with an aim to forming the type of flexible structures used to measure and modulate the contractile forces of cells in tissue engineering experiments. The successful replication of grating structures is demonstrated with feature sizes as low as 100 nm and an analysis of certain processing conditions that facilitate and enhance the accuracy of this replication is presented. The results are benchmarked against an optical storage media grade polycarbonate.

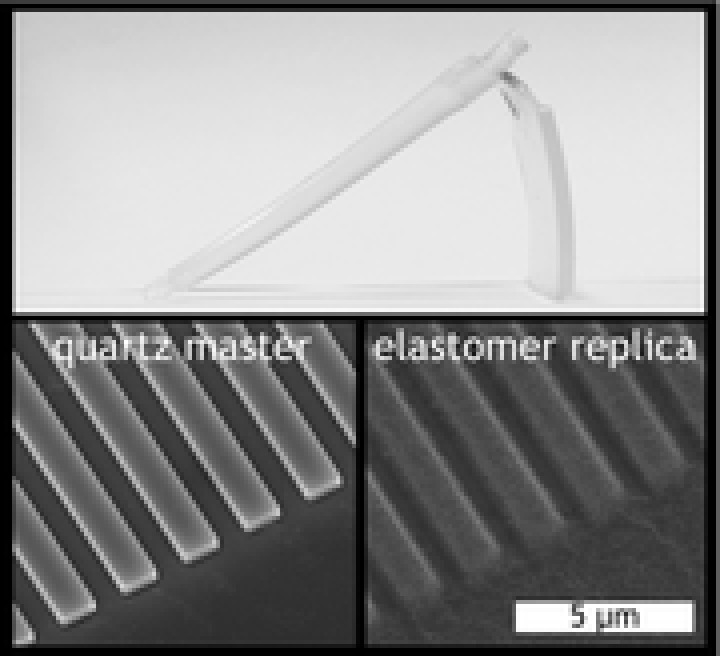

## 1 Introduction

Amongst the ever‐growing toolbox of micro‐ and nanofabrication techniques, polymer replication‐based processes continue to attract attention due to the conceptual simplicity, reproducibility, and great variety of often cheap materials that can be used. Cost and time saving advantages are further provided by high throughput replication techniques such as roll‐to‐roll nanoimprint lithography^1^ and injection molding.^2^ Injection molding is an industrially established technique regularly used to replicate the nanoscale features of optical media and has been proven capable of realizing features as small as 5 nm.^3^ The process involves the forcing of molten thermoplastic polymer into a mold cavity under high pressure where it rapidly cools to below its glass transition temperature (*T*_g_) and solidifies. The tool then opens and the newly formed bulk part is ejected to complete the automated process. Micro‐ and nanopatterning is achieved by patterning one or more of the cavity walls, often through the use of interchangeable inserts, sometimes referred to as *stampers* or *inlays*, which can be produced in a number of ways and are the subject of much of the literature in the field.^2^, ^4^, ^5^, ^6^, ^7^

Elastomers are a family of polymers which exhibit greater mechanical flexibility than other types of polymer due to the fact that their glass transition temperatures lie below room temperature. Their flexible and elastic properties see them find use in many commercial applications, including clothing, cabling, and automotive parts. They also play an important role as microstructured materials in areas of study like cell biology,^8^, ^9^, ^10^ microfluidics,^11^ and dry adhesion.^12^ Thermoplastic elastomers (TPE) form a subset of the group and are useful in situations where the combination of rubber‐like properties and application to thermoplastic processing techniques is advantageous. Although applicable to many industries and applications, the interest in TPEs in this work stems primarily from the use of other elastomers like polydimethylsiloxane (PDMS) and other soft materials such as hydrogels in cell biology research. Flexible pillars offer a model for in vitro substrate rigidity that can be tuned by the height of the pillars and mechanical properties of the polymer.^13^, ^14^, ^15^ When cells adhere to such pillars they exert sufficient force to bend them and, by measuring the extent of the bending, it is possible to determine the magnitude of this force if the height and mechanical properties of the pillars are known. As well as being a measurement tool, these pillars cause changes in cell morphology and spreading and can influence the lineage commitment of stem cells through cues mediated by both substrate stiffness^16^, ^17^ and micro‐ and nanoscale surface structure.^18^, ^19^

If experiments such as these are to be up‐scaled it is desirable to use a high throughput technique like injection molding that can produce thousands of samples per day rather than PDMS casting which can take several hours to form a single replica. When the mechanical properties of TPEs are similar to these currently used materials (as in the work of Fu et al.^13^ where the Young's Modulus of PDMS was 2.5 MPa) then injection molding may offer a route to high throughput production of samples that will enable this type of work to be done on a larger scale.^15^

**Figure 1:**
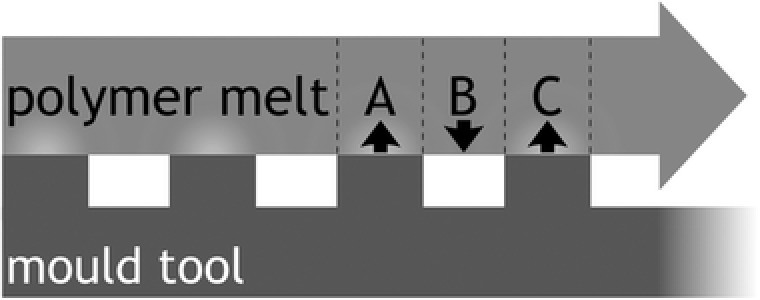
Schematic showing regions of differential cooling as polymer flows over the mold surface during the filling of a microstructured mold. Regions A and C make contact with the mold before region B and so experience a faster rate of cooling which can induce stress in the material. Image adapted from Pranov^20^ 2006.

The elastic nature of TPEs, while a desirable property in many situations, is also a limitation when it comes to trying to manipulate their shape in a permanent way. Any externally applied deformation, be it a simple push or pull, will cause stress in the material which will result in a return toward its original shape when the external influence is removed. In the case of a replication‐based process like embossing or injection molding this behavior is manifested in a tendency for newly formed structures to relax and loose some or all of their applied shape to this elastic stress. In his doctoral thesis,^20^ Pranov compared the success of replicating microstructures by hot embossing and injection molding with the elastomer Tecoflex (Thermedics Inc.—now Lubrizol), a thermoplastic polyurethane (TPU). The embossing results were more successful than those achieved with injection molding, leading him to hypothesize that the injection molded features were subject to greater relaxation after demolding than those on the embossed products due to the fact that the injection molding process induced a greater stress in the material. To test this, he attempted to reduce the stress by lowering the injection speed and found that this did indeed improve results, though not to the extent that the results matched the quality of those made by embossing. He also found that the addition of a nonadhesive coating to the tooling surface to be beneficial to replication, consistent with other results,^4^, ^21^ including those reported by Yoon et al.^22^ Yoon et al. also identified increasing tool temperature as a means to improve microstructure formation with a TPU (Texin 990, Bayer). Their work, however, found a contradictory relationship with injection speed to that reported by Pranov where faster speeds would slightly enhance replication, an effect which they explained by sheer thinning. In the case of sheer thinning, viscosity is decreased by an increase in sheer stress (applied here by increased injection speed) which may improve filling, but it would be expected that this increased stress would induce greater elastic relaxation after removal from the mold. Perhaps the increased mold temperature (*T*_w_) (Yoon used 66 °C vs the manufacturer's recommended 16–43 °C—Pranov used 25 °C) was sufficient to counteract such effects by inducing a viscosity reduction independent of sheer thinning. Looking at the schematic in Figure 1, as injected polymer fills a micropatterned mold there will be occasions where regions of polymer which are touching the inlay surface (A and C) are adjacent to regions which are not (B). The differing cooling rates experienced by these neighboring regions can also induce stress, but Yoon's higher tool temperature could have mitigated this effect (as could the slower cooling rates provided by heat retardant tooling if it were used).

It is not possible to draw direct comparisons between these two works as they used different polymers, equipment and tooling, but they offer a guide to further investigations and a context in which to evaluate future results. They both agree on the widely accepted benefit of nonadhesive surface coatings and point to injection speed as a key parameter for the success of injection molding microstructures with TPU, while Yoon points to tool temperature as another variable worth considering.

This paper is primarily concerned with an assessment of the ability of a TPU to replicate micro‐ and nanostructures by injection molding by means of a study into the effects of injection speed and feature size on the replication fidelity of this polymer. Before the results of this study are presented, fabrication methods are discussed and polycarbonate (PC) is included as a benchmark for replication fidelity against which to compare the performance of the elastomer due to its excellent performance in earlier works.^4^, ^23^ Changing the tool temperature was not assessed except to establish that there were practical limitations that prevented it from being altered by any significant amount. Finally, we draw conclusions about the performance of the TPU and if it can be used, in conjunction with the coated polymer tooling, to fabricate elastomeric microstructures for cell engineering and other applications.

## 2 Experimental Section

### 2.1 Master Fabrication

Two master stamps were fabricated in quartz by electron beam lithography (EBL) (Vistec VB6 UHR EWF 100 kV beam writer, PMMA resist), metal lift‐off in 50 °C acetone (120 nm NiCr) and dry etch (CHF_3_/Ar, Oxford 80+ RIE etch tool) and coated with a nonadhesive fluorosilane layer, trichloro(1H,1H,2H,2H‐perfluorooctyl)silane (TPFS), applied by vapor deposition to a surface activated by oxygen plasma. Surface roughness of both etched and unetched quartz surfaces was <2 nm (R_a_) and the taper angle of the etch was 3°–5° from vertical. The stamps contained a series of grating patterns all measuring 500 μm in length etched to a depth of 302 nm on one stamp and 592 nm on the other (standard deviation = 5 nm for both). The grating patterns had line widths of 100, 250, 500, and 1000 nm and gap:ridge ratios of 4:1, 3:1, 2:1, and 1:1, respectively.

### 2.2 Pattern Transfer to Inlays

The grating patterns were transferred from the quartz stamps to SU‐8 on Cirlex by UV‐NIL embossing using a custom built apparatus as described in an earlier work^4^ resulting in two Cirlex/SU‐8 inlays which were subsequently coated with a nonadhesive coating comprising 15 nm SiN_x_ (induction coupled plasma deposition) and a monolayer of vapor deposited TPFS.

### 2.3 Injection Molding

Injection molding was performed with an Engel Victory 28 hydraulic injection molding machine (max injection pressure = 2200 bar, max clamping force = 280 kN, max shot volume = 20 cm^3^, max injection speed = 52 cm^3^ s^−1^). Inlays were inserted into a frame in the tool which was designed to produce parts measuring ≈25 × 25 × 2 mm^3^ with one patterned face corresponding to the side of the mold cavity where the inlay was situated.

Polycarbonate was molded with the following conditions based on the manufacturer's guidelines: *T*_m_ = 280 °C, *T*_w_ = 80 °C, *v*_i_ = 50 cm^3^ s^−1^. TPU was molded with the following conditions: *T*_m_ = 190 °C and 200 °C, *T*_w_ = water cooled to room temperature (20–25 °C), *v*_i_ = 0.9–18.0 cm^3^ s^−1^. Despite the importance of *T*_w_ discussed above it was, unfortunately, not possible to vary this to a lower value due to the nature of the water cooling system or to a higher value for reasons discussed below.

### 2.4 Metrology and Characterization

A combination of optical microscopy, scanning electron microscopy (SEM), and atomic force microscopy (AFM) were used to characterize stamps, inlays, and replicas. Optical microscopy and SEM provided largely qualitative information while AFM was used to determine values for the heights and periodicity of fabricated grating structures. Narrow (aspect ratio 8:1) AFM scans were obtained and flattened in Gwyddion SPM data analysis software (www.gwyddion.net), then analyzed in Matlab where height values were obtained by peak‐to‐peak histogram analysis and period values by FFT analysis. For each distinct experimental condition at least four scans over at least two different samples were averaged to generate mean values and standard errors for subsequent graphical analysis, except for inlays and the quartz masters for which only single samples existed.

### 2.5 Evaluation of Materials

The TPU Tecothane soft AR62A was obtained as a gift from Lubrizol for this investigation. For industrial applications the hardness of an elastomer is characterized by a metric known as the Shore hardness which measures the material's resistance to indentation. An approximate conversion from Shore hardness to Young's modulus is given by Equation (1), 24

(1) $$E=\frac{0.0981\left(56+7.62336S_{A}\right)}{0.137505\left(254-2.54S_{A}\right)}$$

where *E* is the Young's modulus in MPa and *S_A_* is the Shore A hardness (the A denotes one of several different Shore scales). This equation was used to compare the mechanical properties of Tecothane soft AR62A to the type of PDMS used in the instructive biology papers with the flexible pillars.^13^ Its Shore A hardness of 62 corresponds to an approximate Young's modulus of 3.91 MPa which is close to the ≈2.5 MPa for the PDMS in Fu et al.^13^

An initial injection molding test was performed with a nanopatterned polymeric inlay containing 100 nm diameter pits to form pillars. Upon ejection, it was possible to observe the nanopattern mediated optical refraction that would be expected from an engineering grade thermoplast like PC. Sadly, this effect disappeared within a few seconds, likely due to the polymer relaxing to a more energetically stable conformation. Subsequent inspection by AFM and SEM showed no evidence that nanostructures had ever been present, leading to the initial conclusion that it was not possible to produce long‐lived pillar structures of around 100 nm in size with this polymer. A systematic investigation was then undertaken to establish what the minimum feature size that can reliably be produced in the TPU material investigated. The primary variable for consideration in pursuit of this minimum feature size was chosen to be injection speed due to the apparent importance of this parameter as discussed above. Although the effects of temperature conditions were also likely to be important, certain equipment limitations, which are discussed in due course, prevented these from being altered by any significant amount.

## 3 Results and Discussion

### 3.1 Polycarbonate as a Benchmark Material

**Figure 2:**
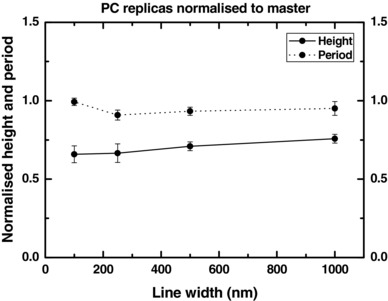
Normalized heights and period of injection molded PC lines. Gap ratio = 4:1 (period = 5 × line width), heights normalized to master stamp (592 nm height for 1000 nm line width, 302 nm height for the others). *T*_m_ = 280 °C, *T*_w_ = 80 °C, *v*_i_ = 50 cm^3^ s^−1^.

Due to the aspect ratio of the narrower features it was not possible to directly assess all trench depths on the inlay by AFM. Additionally it was deemed desirable to have results from an exemplary polymer against which to compare the performance of the TPU. PC (Makrolon OD 2015) was chosen due to its proven ability to faithfully fill nanostructures,^23^ even at ultrahigh aspect ratios,^4^ and, although it has a tendency to stretch at high aspect ratio, it was the best available option for the task at hand. PC was injection molded against the SiN_x_/TPFS coated Cirlex/SU‐8 inlays and the resulting parts measured by AFM to obtain the plots in Figure 2. The plot shows that the depth of filling was consistent at about 75% of master height and the periodicity of the structures was reliably preserved. Surface roughness analysis of AFM scans also revealed that the master's R_a_ was reliably preserved below 2 nm.

### 3.2 Replication by Injection Molding with TPU

**Figure 3:**
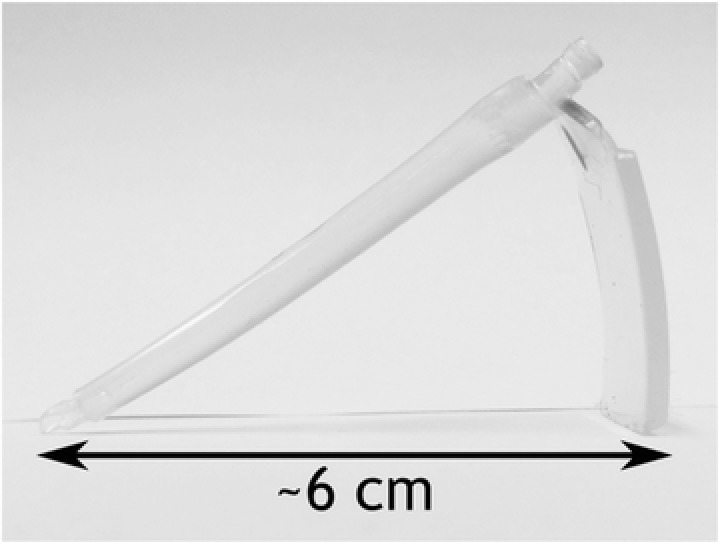
Injection molded parts made from Tecothane soft AR62A.

Observation of the bulk shape of injection molded TPU parts reveals a significant amount of bending in their resting states (see Figure 3) which is indicative of the elastic stress built up within the material. This is a consequence of the differential cooling experienced in the tool in which one side of the part is cooled against the tooling steel and the other against the Cirlex inlay which conducts the heat of the molten polymer away much more slowly.

### 3.3 The Effect of Line Width

**Figure 4:**
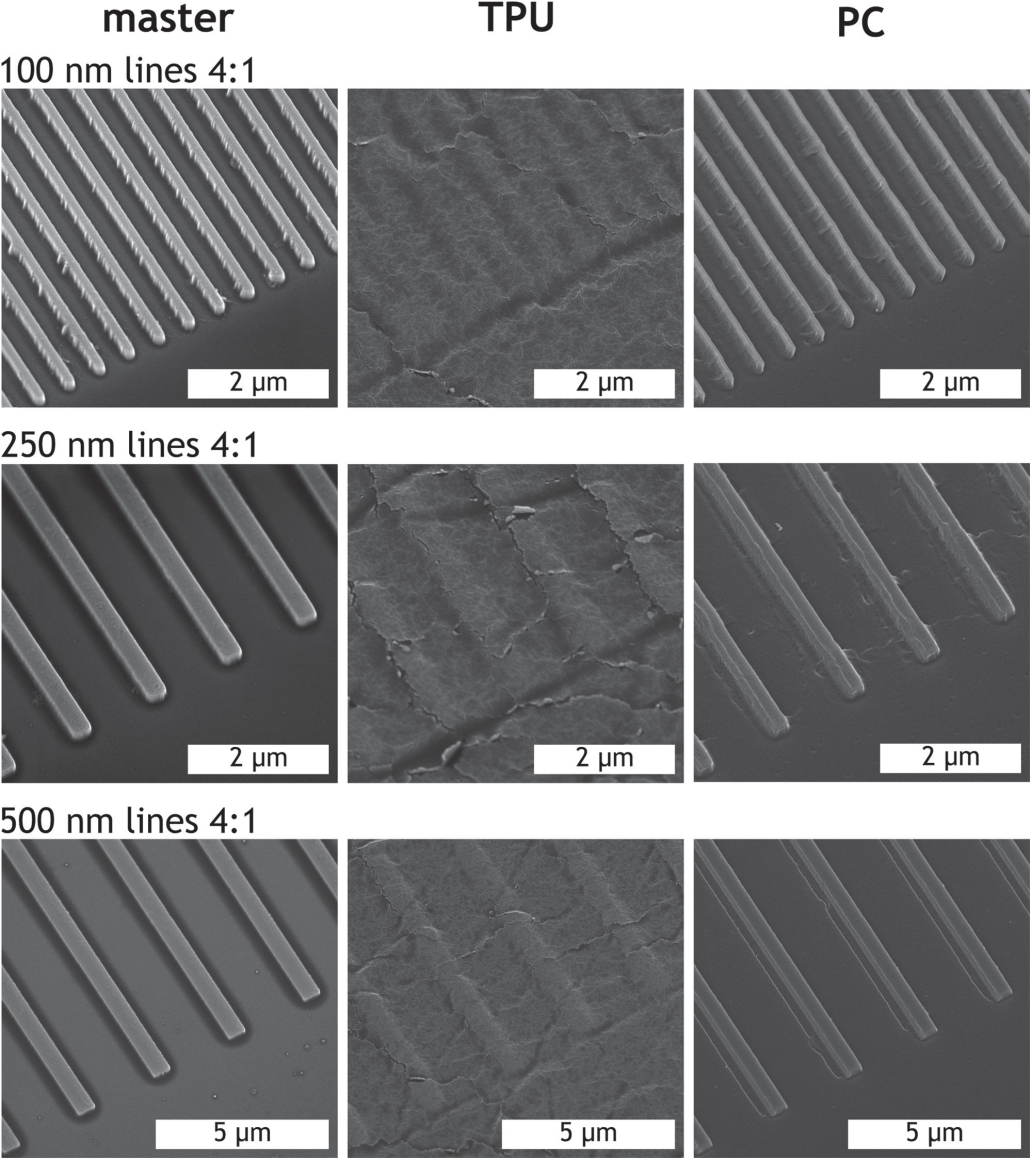
SEM images of master and corresponding replicas made of TPU and PC with gap ratio 4:1. Viewing angle = 30°.

SEM imaging of injection molded TPU parts (Figure 4) revealed that reproduction of the microstructures was achieved, but all features displayed significant rounding at the corners compared to the 302 nm deep master stamp and PC replicas and show signs of lateral relaxation. Furthermore, the thinnest ridges (100 nm) were very poorly defined and had a tendency to bunch together. Additional AFM data to further elucidate the nature of these difficult‐to‐define features is provided in the Supporting Information. A mottled surface topography independent of the intended patterning can also be observed on the TPU replicas. This is thought to be the Au/Pd coating applied for SEM imaging becoming cracked on the flexible sample during handling as there is no evidence of this on uncoated samples imaged by AFM; indeed, the AFM scans reveal that the masters' R_a_ values were preserved below 2 nm.

**Figure 5:**
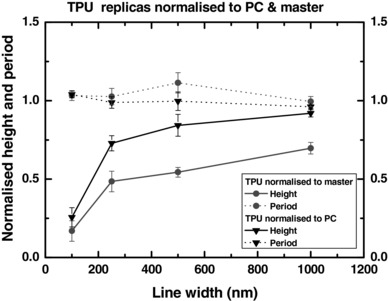
Heights and periodicity (normalized to stamp and PC replicas) of features of different line widths produced in TPU by injection molding. Master height = 592 nm for 1000 nm line width, 302 nm for the others. *T*_m_ = 190 °C, *T*_w_ = 20–25 °C, *v*_i_ = 0.9 cm^3^ s^−1^, gap:ridge ratio = 4:1.

AFM measurements were used to characterize injection molded TPU parts in terms of the height and lateral periodicity of the structures produced. Figure 5 shows how these dimensional quantities, normalized to both the quartz masters and the corresponding PC replicas, are affected by diminishing feature size. The data were normalized to provide a more direct comparison between master, TPU, and PC. Despite the lack of edge definition observed in Figure 4, filling of TPU is not significantly reduced at the largest (1000 nm) line width compared to PC, but does begin to drop off, moving below 75% at line width = 250 nm and falling very quickly toward zero, reaching 25% by line width = 100 nm. It is interesting to note that the slight bunching of lines visible in the SEM images for the smallest grating does not result in substantially larger error bars for the periodicity. This suggests that this effect is not pronounced over the bulk area of the grating pattern.

### 3.4 The Effect of Gap Size

**Figure 6:**
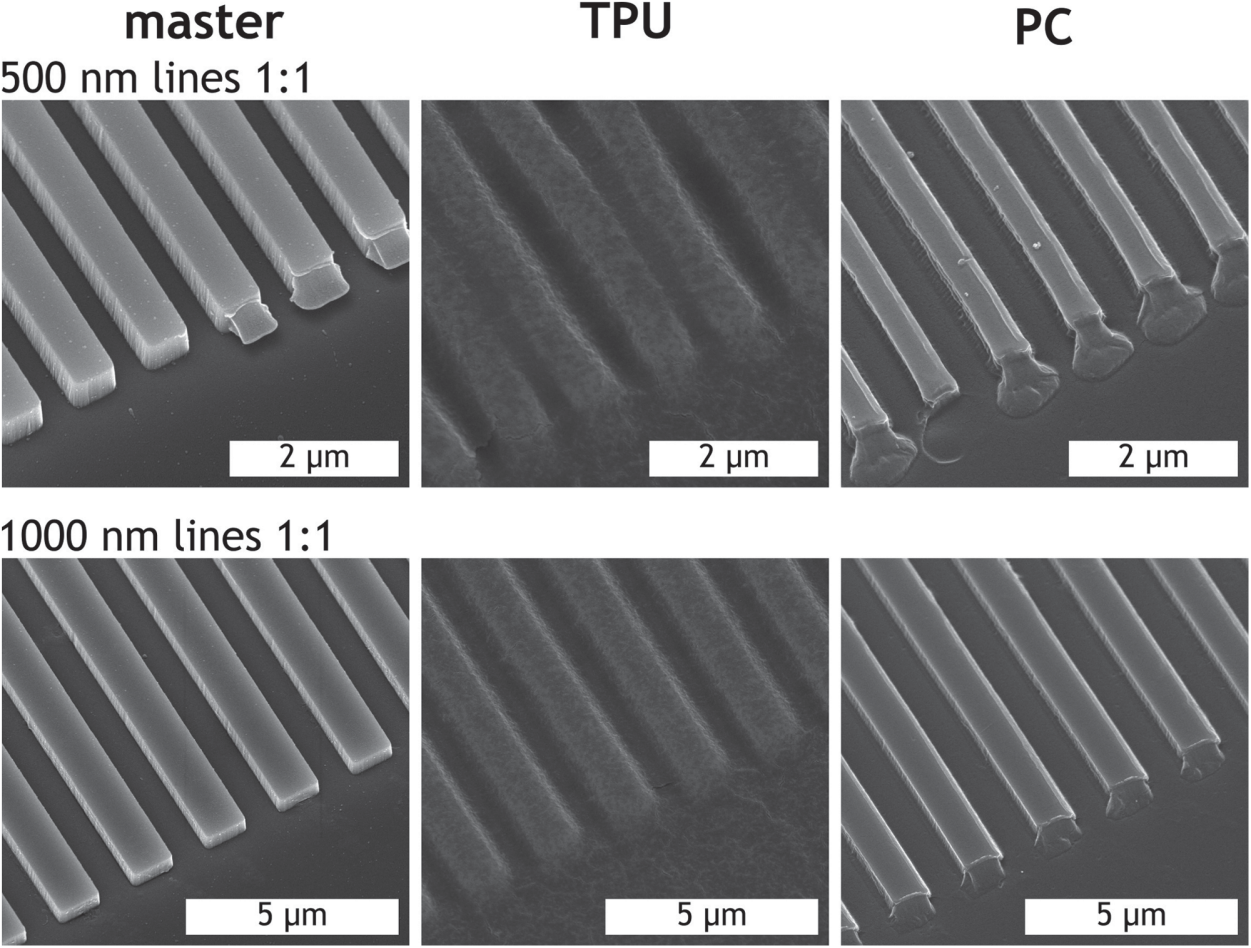
SEM images of master and corresponding TPU and PC replicas with gap ratio 1:1. Viewing angle = 30°.

The other important dimensional variable to consider is the gap between the features. Due to the apparent relaxation of TPU, it seemed likely that features spaced closer together might be less well defined compared to those spaced further apart. Figure 6 shows comparative SEM images of the 592 nm deep quartz master alongside TPU and PC replicas at two line widths: 1 μm, where the replication at a gap ratio of 4:1 is relatively reliable, and 500 nm, where the filling depth of TPU begins to fall away from that of PC. In addition to rounded corners, TPU replicas show signs of ridges clumping together with their neighbors as was observed for 100 nm lines at gap ratio = 4:1 (gap size = 400 nm). This comparison may suggest that it is the reduction of the gap size more than that of the ridge width which is the limiting factor. Another phenomenon noticeable in this figure is the presence of objects stuck to the ends of ridges on the master stamp and corresponding features at the ends of lines in the PC replicas. On the stamp these are pieces of SU‐8 displaced during the UV embossing process which adhered upon curing and separation. The negative image of these missing volumes of SU‐8 is present in the pattern transfer to the inlay and is therefore observable on PC replicas, but not TPU replicas which fail to reproduce these more subtle structures.

**Figure 7:**
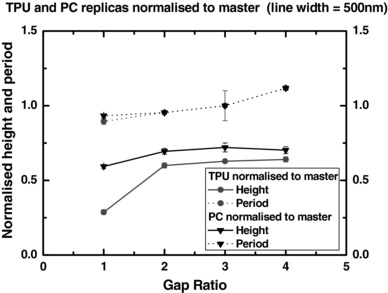
Effect of diminishing gap ratio on the replication of 500 nm gratings by injection molding with PC and TPU. Master height = 592 nm, *T*_m_ = 190 °C, *T*_w_ = 20–25 °C, *v*_i_ = 0.9 cm^3^ s^−1^.

Figure 7 compares the normalized height and periodicity of 500 nm TPU ridges across the range of gap:ridge ratios with PC results provided for comparison. Although, as expected, the TPU replicates the heights of structures less well than PC overall, the performance of both polymers is relatively consistent until the gap:ridge of 1:1 with the TPU showing a much more noticeable drop. Again, it appears that the bunching of the ridges with the smallest gap does not correspond to an increased error in the periodicity of gratings in the bulk.

### 3.5 The Effect of Injection Speed

**Figure 8:**
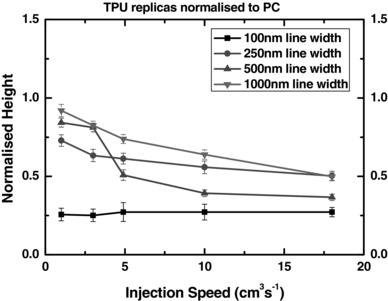
Effect of injection speed on heights (normalized to PC replicas originating from the 592 nm master for 1000 nm lines and the 302 nm deep master for the others) of TPU features for four line widths. *T*_m_ = 190 °C, *T*_w_ = 20–25 °C, gap ratio = 4:1.

It has been shown that injection speed, a parameter directly and monotonically related to injection pressure (injection pressure is not a parameter that can be specified on the molding machine used in this work, but could be altered indirectly by changing the injection speed), is an important parameter in the formation of microstructures in elastomers.^20^, ^22^ The plots in Figure 8 show how injection speed affects feature heights for gap ratio = 4:1. Periodicity was maintained consistently (data not shown). For all feature sizes except 100 nm ridges we see a clear improvement in replication as injection speed is reduced. This is consistent with results observed by Pranov^20^ which indicate that the replication of height is indeed improved by a reduction in injection speed and suggest that at zero injection speed, analogous to an embossing process, height replication would be even better and may even improve for the smallest features.

### 3.6 The Effect of Temperature Conditions

It was mentioned earlier that tool and melt temperatures may play a role in enhancing the replication quality of TPUs. In all of the above results, the temperatures were set as per the manufacturer's guidelines with melt at 190 °C and the tool water cooled to between 20 and 25 °C. A set of results was obtained with the melt temperature raised to 200 °C (data not shown), but this showed no significant variation from the results obtained at 190 °C across all metrics studied above. Attempts were also made to use a raised tool temperature but upon heating from 30–50 °C any attempt to run a cycle resulted in the sprue breaking part of the way along its length during the normal manual part removal resulting in a time‐consuming process to remove said sprue section from the tool. While this could be due to the polymer being in a more brittle phase in this temperature range, the precise reasons for this behavior remain unclear but and it ultimately served to prevent any investigation of this parameter with the available equipment.

## 4 Conclusions

Our established polymer tooling solution^4^, ^23^ has been adapted with relative ease to allow for high throughput replication of elastomeric micro‐ and nanostructures. Although molded parts had to be manually removed after every cycle, which negates the possibility of a fully automated process, it is still relatively fast compared to other replication‐based microfabrication techniques.

Elastomeric micro‐ and nanopatterns were fabricated with success although corner definition was universally inferior to that of PC. Compared to PC, the filling depth of TPU was not significantly worse at for the largest feature sizes (1000 nm line width at 4:1 gap ratio), but did decrease with a tendency towards zero as these values diminished.

A clear value for the minimum attainable feature size for Tecothane soft AR62A was not obtained; however, it is apparent that clarity of replicated features begins to become compromised as the size of ridges or the gaps between them descend to around 400 or 500 nm. While it is difficult to define effects like the rounding of corners in a quantitative way, if one considers a corner to be itself a *small* feature then our quantitative assessment of height replication at diminishing line widths can serve as an analogous guide for the limitations of this polymer for accurate replication of sharp corners.

Reduced injection speed was shown to clearly improve filling of all except the smallest (100 nm) line width, but it may be the case that a near zero injection speed, analogous to embossing, may improve height replication for features of that size and even smaller. A marginal (10 °C) increase in melt temperature showed no significant improvement to height replication and it was not possible to investigate tool temperature with the available equipment, although it is suspected that raising this would likely improve height replication (i.e., the prevention of elastic relaxation after mold cavity filling and subsequent demolding) through the mitigation of sheer thinning effects.

Having established the limitations with Tecothane soft AR62A under certain fixed conditions (*T*_m_, *T*_w_, equipment), it would next seem sensible to try to vary these in a systematic way to further push the boundaries of what is possible with this material. This might not be possible with the injection molding machine used in this work, but one with a lower range of injection speeds and a tool designed specifically for elastomers may produce better results.

The results presented here can be used to inform the design and fabrication attempts for cell force application structures in the future. In doing so it would be possible to establish a platform by which to rapidly fabricate large numbers of samples which could be used in experiments to modulate the rigidity of substrates and measure the forces exerted by cells instead of PDMS pillars used previously.^8^, ^13^ While a fully automated process was not achieved, the throughput of micropatterning the TPU Tecothane soft AR62A by injection molding using coated Cirlex/SU‐8 inlays is a promising method for fabricating large numbers of micropatterned elastomeric samples for whatever applications may require them.

## Supporting information

As a service to our authors and readers, this journal provides supporting information supplied by the authors. Such materials are peer reviewed and may be re‐organized for online delivery, but are not copy‐edited or typeset. Technical support issues arising from supporting information (other than missing files) should be addressed to the authors.

Supplementary
