# Supplementary material for: Injection Molding Micro‐ and Nanostructures in Thermoplastic Elastomers
Source: Macromol Mater Eng. 2016 May 6;301(8):964–71. doi: 10.1002/mame.201600011 (PMC6563430; doi:10.1002/mame.201600011)
Supplement: Supplementary file 1 — Supplementary [file MAME-301-964-s001.pdf]

Copyright WILEY-VCH Verlag GmbH & Co. KGaA, 69469 Weinheim, Germany, 2016.

Supporting Information

for *Macromol. Mater. Eng.,* DOI: 10.1002/mame.201600011

Injection Molding Micro- and Nanostructures in Thermoplastic Elastomers

John M. Stormonth-Darling, Anwer Saeed, Paul M. Reynolds, Nikolaj Gadegaard*

Supporting information for
Injection moulding micro- and nanostructures in thermoplastic elastomers

John M. Stormonth-Darling[†], Anwer Saeed[†], Paul M. Reynolds and Nikolaj Gadegaard*

[†]These authors contributed equally in the preparation of this paper.

Height distribution data from AFM scans

In addition to the complimentary quantitative graphical data and qualitative SEM data presented in the main paper, it may be useful to the reader to see more information that further elucidate hard-to-define phenomenon such as the rounding of edges in replicated TPU structures. To this end we provide a selection of height distribution data obtained from AFM scans. The data is normalised in both axes so that differences in absolute height and sharpness of peaks do not interfere with the reader's appreciation of the differences in height distribution. Representative scans have been used in all cases.

An ideal grating with vertical edges (and no AFM tip convolution) would appear on a height histogram as two delta functions indicating the tops and bottoms of lines/trenches with all zero values in between. The less well defined the grating structure (i.e. more rounded the corners), the less distinct the high and low peaks will be amongst the noise of other height values around and between them.

The following histograms are split into sections in a similar way to the division of sections in the main paper.

Line width

Normalised histograms are presented of the height distribution data for grating patterns of 5 different line widths replicated in TPU alongside the quartz master and benchmark PC before a final graph comparing TPU replicas of all sizes.

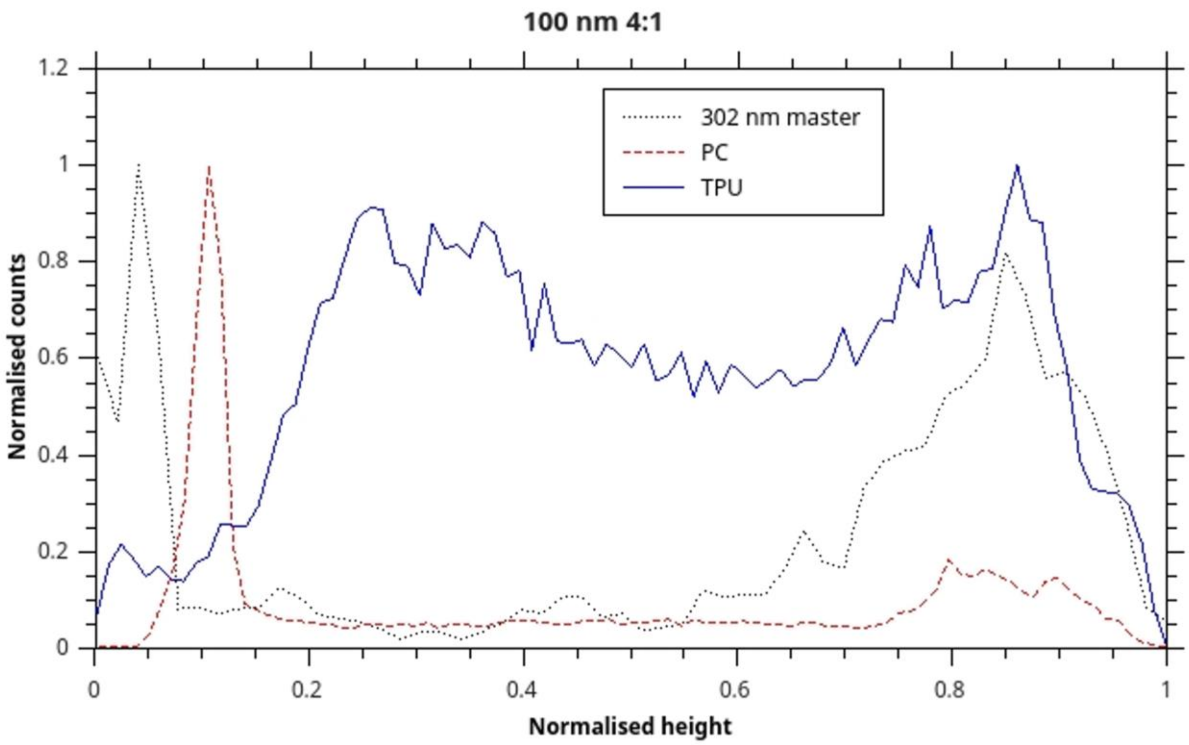
100 nm 4:1
302 nm master
PC
TPU
Normalised counts
Normalised height
0
0.2
0.4
0.6
0.8
1
1.2

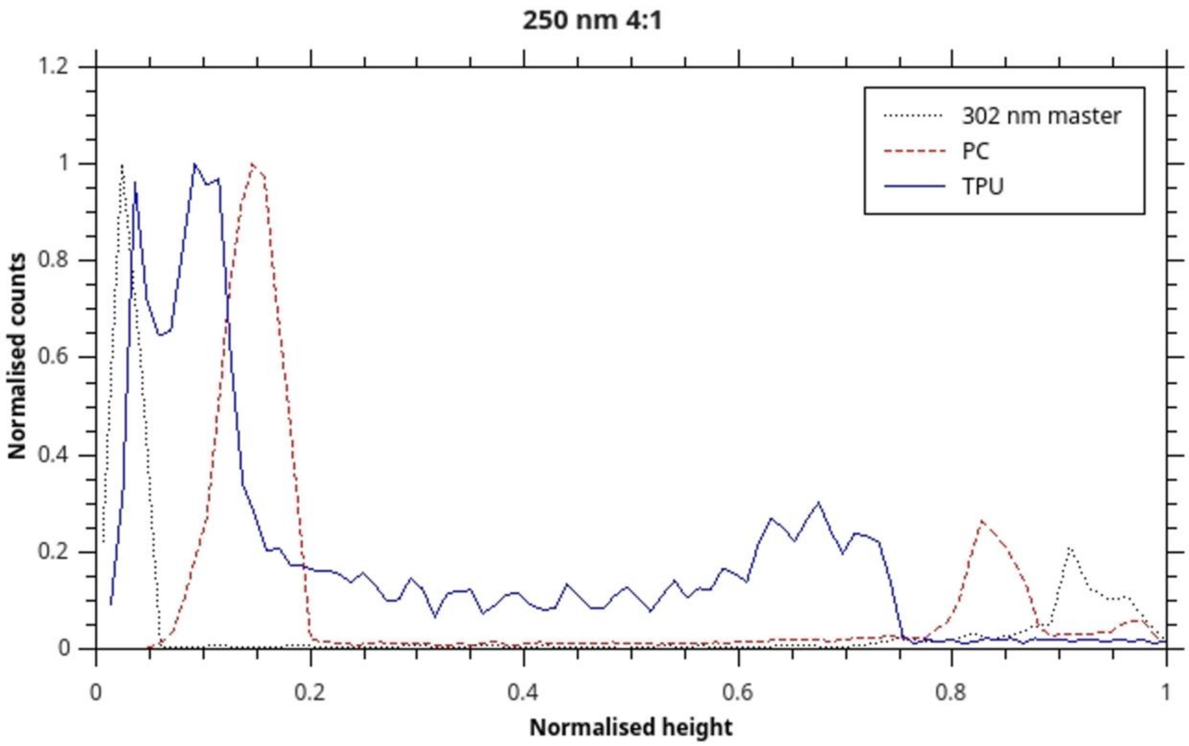
250 nm 4:1
302 nm master
PC
TPU
Normalised counts
Normalised height
0
0.2
0.4
0.6
0.8
1
1.2

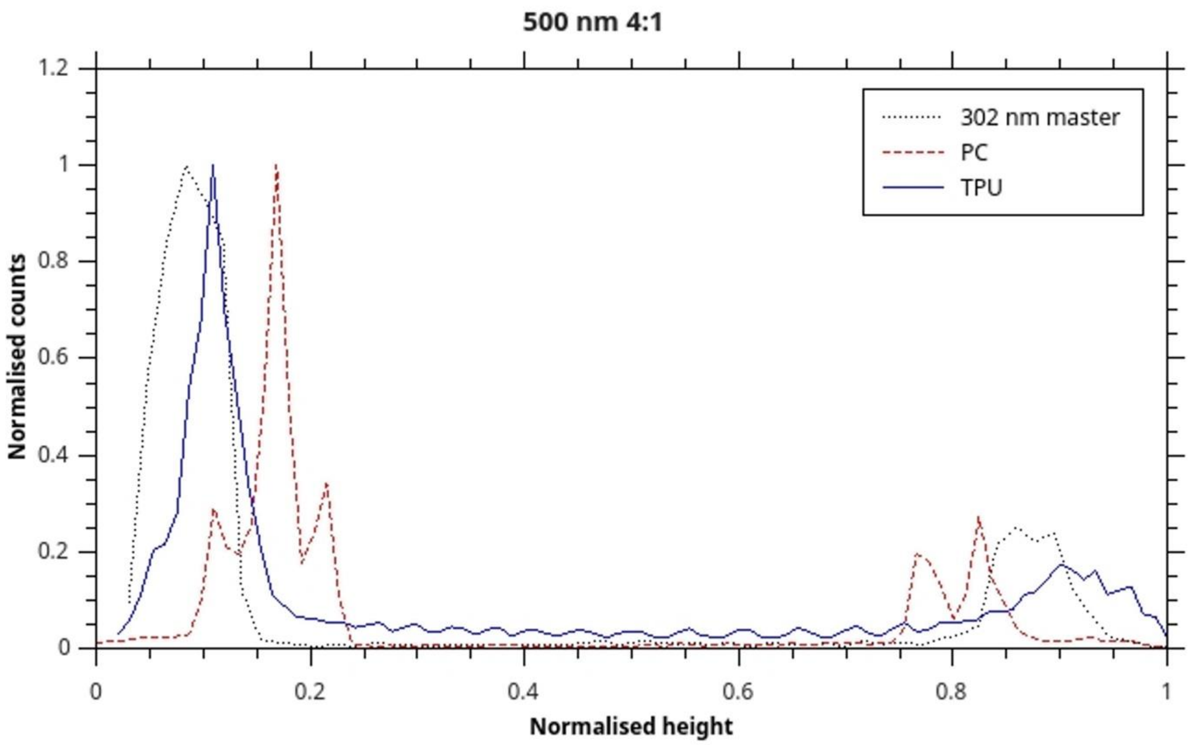
500 nm 4:1
302 nm master
PC
TPU
Normalised counts
Normalised height
0
0.2
0.4
0.6
0.8
1
1.2

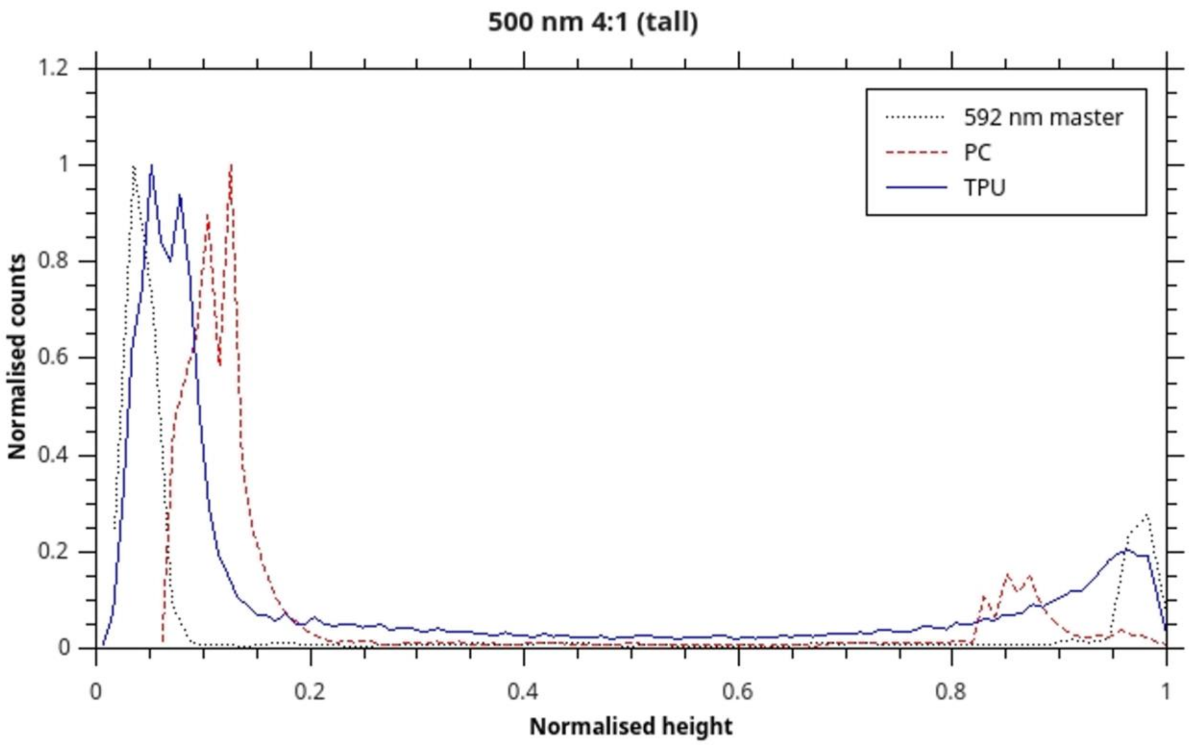
500 nm 4:1 (tall)
592 nm master
PC
TPU
Normalised counts
Normalised height
0
0.2
0.4
0.6
0.8
1
1.2

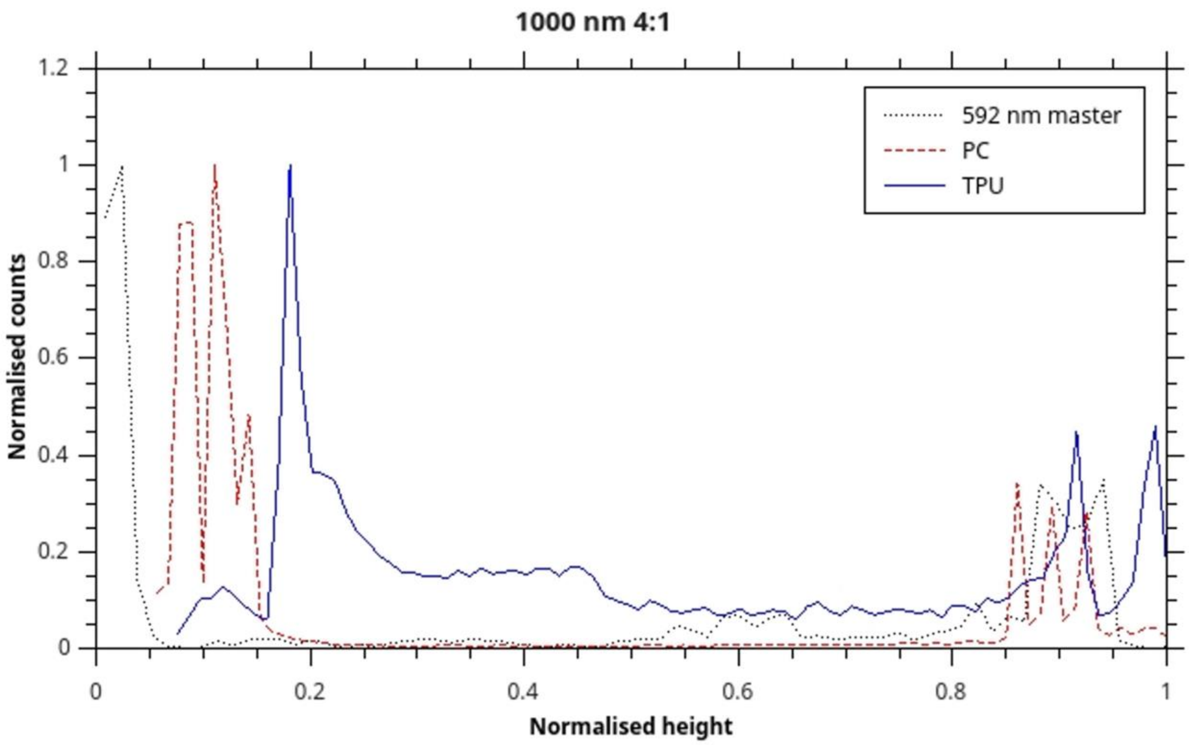
1000 nm 4:1
592 nm master
PC
TPU
Normalised counts
Normalised height
0
0.2
0.4
0.6
0.8
1
1.2

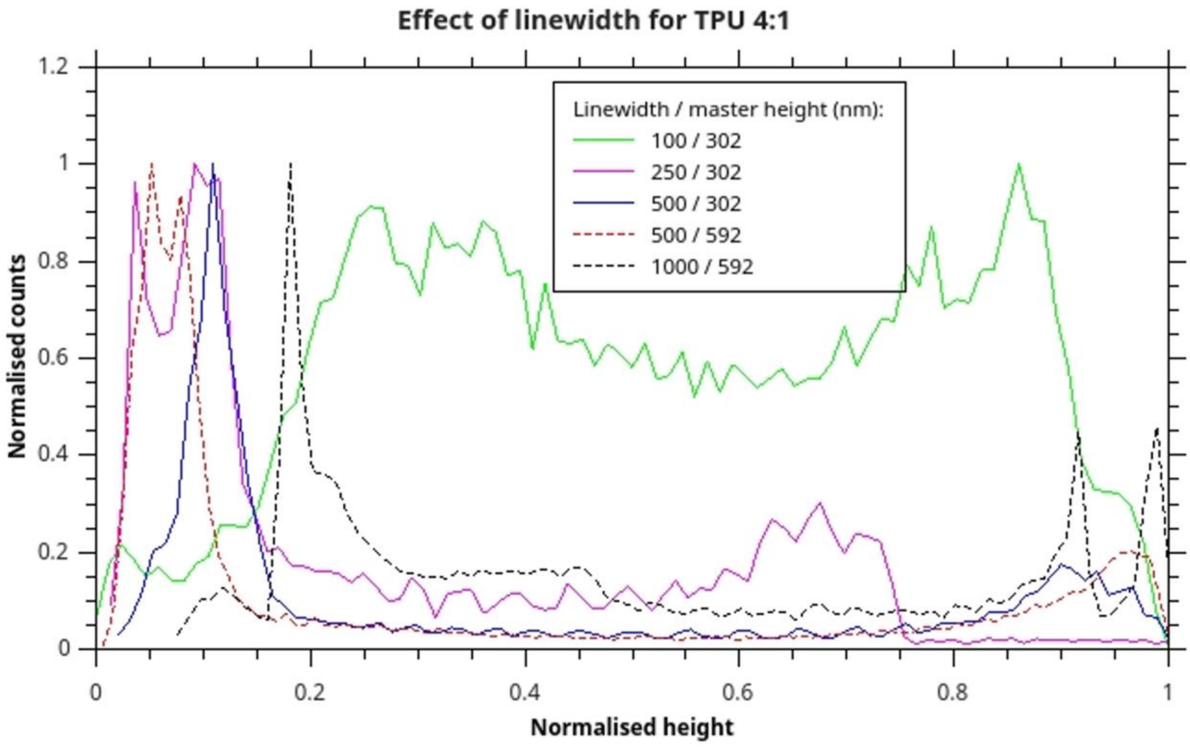
Effect of linewidth for TPU 4:1
Linewidth / master height (nm):
100 / 302
250 / 302
500 / 302
500 / 592
1000 / 592
Normalised counts
Normalised height
0
0.2
0.4
0.6
0.8
1
1.2

Gap ratio

Normalised histograms are presented of the height distribution data for TPU grating patterns of 500 nm and 1000 nm linewidth with 1:1 ridge:gap ratio for comparison with *500 nm 4:1 (tall)* and *1000 nm 4:1* above. Data of equivalent structures on the quartz master and PC replicas is provided on the same plots except in the 3rd graph where 4 different gap ratios for 500 nm linewidth are compared in TPU alone.

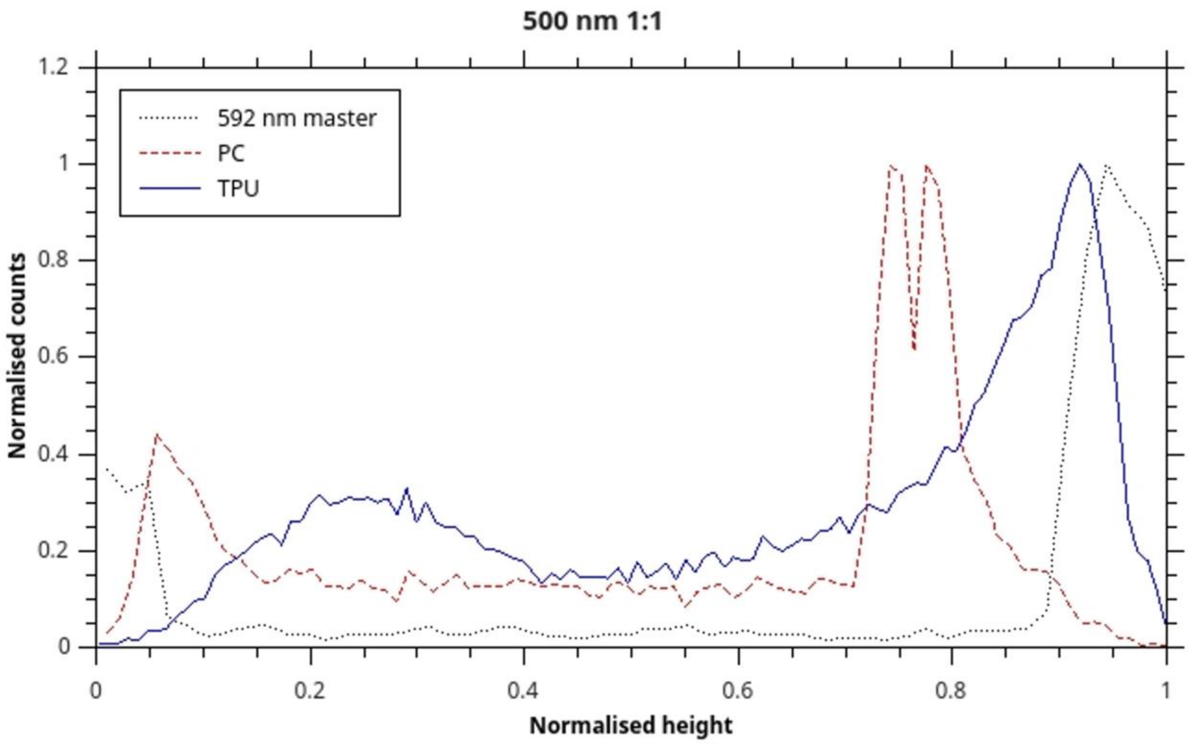

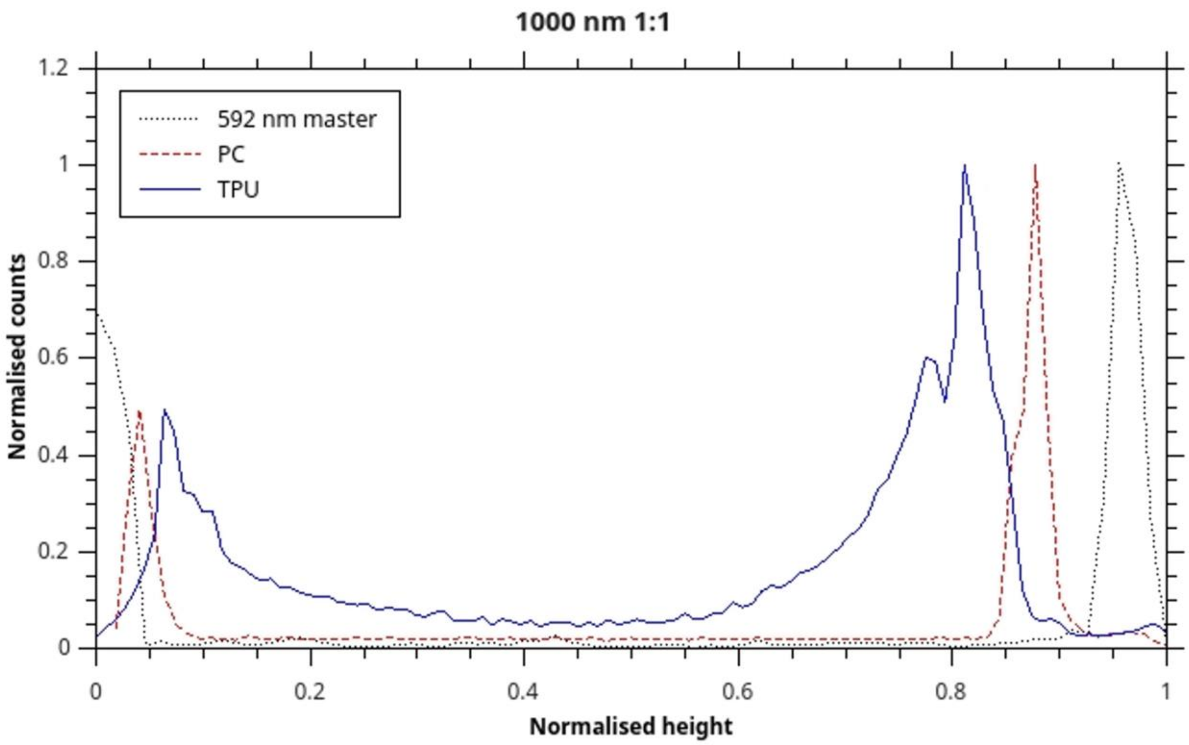

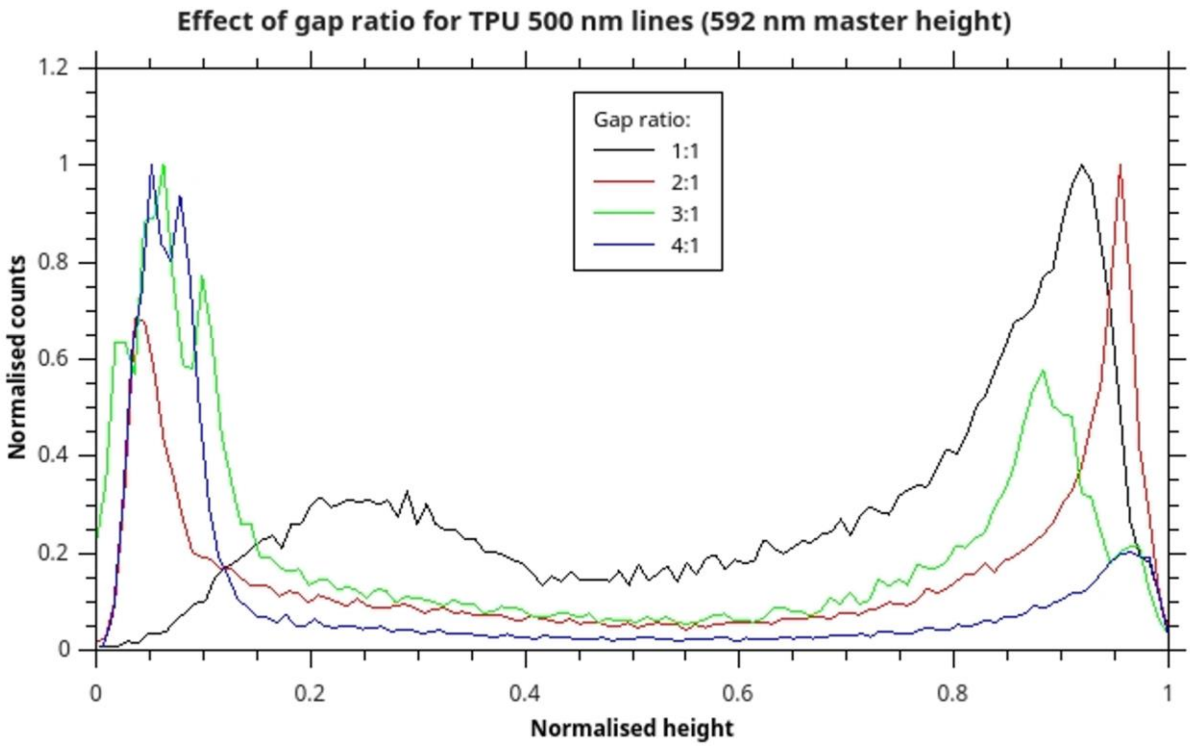

Injection speed

A normalised histograms is presented of the height distribution data for 500 nm grating patterns replicated in TPU at 4 injection speeds.

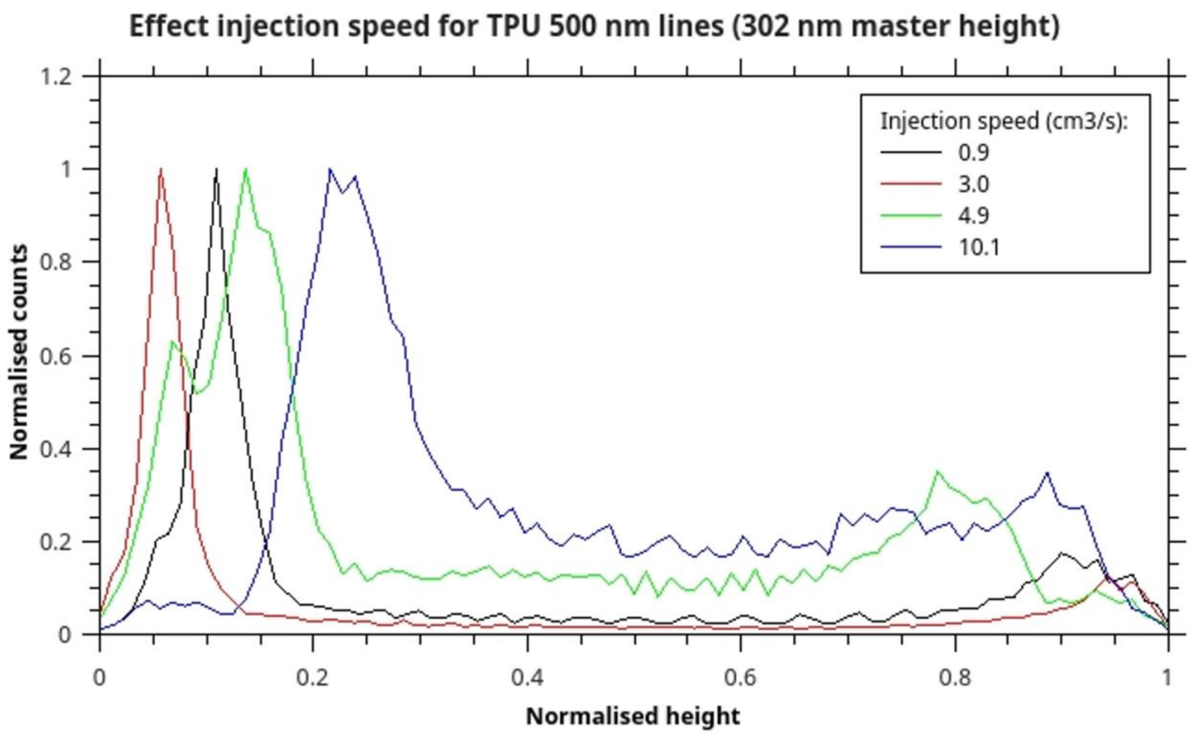

Effect injection speed for TPU 500 nm lines (302 nm master height)
Injection speed (cm3/s):
0.9
3.0
4.9
10.1
Normalised counts
Normalised height
0
0.2
0.4
0.6
0.8
1
1.2
